# Updates of the ERIC recommendations on how to report the results from immunoglobulin heavy variable gene analysis in chronic lymphocytic leukemia

**DOI:** 10.1038/s41375-024-02163-4

**Published:** 2024-02-16

**Authors:** Thomas Chatzikonstantinou, Andreas Agathangelidis, Anastasia Chatzidimitriou, Cristina Tresoldi, Zadie Davis, Véronique Giudicelli, Sofia Kossida, Chrysoula Belessi, Richard Rosenquist, Paolo Ghia, Anton W. Langerak, Frédéric Davi, Kostas Stamatopoulos, ERIC, the European Research Initiative on CLL

**Affiliations:** 1 Institute of Applied Biosciences, Centre for Research and Technology Hellas, Thessaloniki, Greece; 2 Department of Biology, School of Science, National and Kapodistrian University of Athens, Athens, Greece; 3 Department of Molecular Medicine and Surgery, Karolinska Institutet, Stockholm, Sweden; 4 Division of Immunology, Transplantation and Infectious Diseases, IRCCS Ospedale San Raffaele, Milan, Italy; 5 Department of Molecular Pathology, University Hospitals Dorset, Bournemouth, UK; 6 International ImMunoGeneTics Information System (IMGT), Institut de Génétique Humaine (IGH), Centre National de la Recherche Scientifique (CNRS), Université de Montpellier, Montpellier, France; 7 Hematology Department, Nikea General Hospital, Pireaus, Greece; 8 Clinical Genetics, Karolinska University Hospital, Stockholm, Sweden; 9 Division of Experimental Oncology, Università Vita-Salute San Raffaele and IRCCS Ospedale San Raffaele, Milan, Italy; 10 Department of Immunology, Laboratory Medical Immunology, Erasmus MC, Rotterdam, The Netherlands; 11 Department of Hematology, APHP, HôpitalPitié-Salpêtrière and Sorbonne University, Paris, France

**Keywords:** Chronic lymphocytic leukaemia, Genetics research

## To the Editor:

The treatment paradigm of chronic lymphocytic leukemia (CLL) is rapidly shifting from chemoimmunotherapy (CIT) to novel targeted agents. Recent randomized studies have consistently documented the superiority of chemo-free treatment with targeted therapies over CIT in patients with CLL [1–3], rendering the latter either not recommended or a less preferred option, as also reflected in recently published international guidelines [4, 5]. On these grounds, we deem it necessary to introduce some changes to the recommendations by ERIC, the European Research Initiative on CLL [6], on how to report results from immunoglobulin heavy variable (IGHV) gene analysis in CLL in order to reflect the recent advancements in CLL therapy.

More particularly, the aim of this update is to emphasize on the need to inform clinicians about the existing knowledge (or lack thereof) on the predictive value of IGHV gene somatic hypermutation (SHM) status in the era of targeted agents (Table 1 and Supplemental Material). Specifically, ERIC proposes that the laboratory report for patients found by sequencing analysis to carry unmutated IGHV genes should also mention that these patients display shorter progression-free survival when treated with fixed-duration treatment, such as venetoclax plus obinutuzumab compared to IGHV-mutated cases [7]. Finally, the laboratory reports of patients identified as belonging to CLL stereotyped subset #2 should state that this is a prognostically adverse patient group regardless the SHM status [8, 9] while also acknowledging the lack of evidence about the predictive value of subset #2 in the era of targeted therapies.

**Table 1:** Proposed changes in the interpretation section of the previous examples.

Example of the IGHV report	Interpretation
IGHV-unmutated	Following the 98% germline identity cut-off which is used for discriminating CLL cases into the IGHV-mutated or IGHV-unmutated category, this case belongs to the IGHV-unmutated category which is generally associated with adverse prognosis and poor response to chemoimmunotherapy and a shorter progression-free survival when treated with fixed-duration treatments that include venetoclax plus an anti-CD20 antibody compared to IGHV-mutated cases [7].
Subset #2	Following the 98% germline identity cut-off which is used for discriminating CLL cases into the IGHV-mutated or IGHV-unmutated category, this case belongs to the IGHV-mutated category. However, this particular rearrangement belongs to stereotyped subset #2 which is associated with adverse prognosis and poor response to chemoimmunotherapy regardless of the somatic hypermutation status [8, 9]. The predictive value of subset #2 for patients treated with targeted therapies is currently unknown.

### Supplementary information

SUPPLEMENTAL MATERIAL
