# Supplementary material for: Updates of the ERIC recommendations on how to report the results from immunoglobulin heavy variable gene analysis in chronic lymphocytic leukemia
Source: Leukemia. 2024 Feb 16;38(3):679–80. doi: 10.1038/s41375-024-02163-4 (PMC10912022; doi:10.1038/s41375-024-02163-4)
Supplement: Supplementary file 1 — SUPPLEMENTAL MATERIAL [file 41375_2024_2163_MOESM1_ESM.docx]

##### *1. Example of the IG report, IGHV-mutated*

##### Name of the Hospital/Laboratory

**Determination of IGHV gene SHM status**

Date of result: 22/01/2022

Date of sample collection: 09/01/2022

**Patient name:** ***

Diagnosis: CLL

Tissue type: blood

Molecule type: genomic DNA

**Utilized methodology**

PCR amplification of IGHV-IGHD-IGHJ gene rearrangements with leader primers

Genescan analysis

Bidirectional Sanger sequencing [or next-generation sequencing (whichever applies)]

Immunoinformatics analysis: IMGT/V-QUEST [or any specific software for NGS-based IG gene analysis (whichever applies)]

**Result:** A productive IGHV3-23*01/IGHD4-17*01/IGHJ4*02 gene was detected. The rearranged IGHV gene had 96.2% nucleotide identity with the germline sequence of the IGHV3-23*01 gene.

**Interpretation:** Following the 98% germline identity cut-off which is used for discriminating CLL cases into the IGHV-mutated or IGHV-unmutated category, this case belongs to the IGHV-mutated category which is generally associated with favorable prognosis.

Signatures

##### *2. Example of the IG report, IGHV-unmutated*

##### Name of the Hospital/Lab

**Determination of IGHV gene SHM status**

Date of result: 22/01/2022

Date of sample collection: 09/01/2022

**Patient name:** ***

Diagnosis: CLL

Tissue type: blood

Molecule type: genomic DNA

**Utilized methodology**

PCR amplification of IGHV-IGHD-IGHJ gene rearrangements with leader primers

Genescan analysis

Bidirectional Sanger sequencing [or next-generation sequencing (whichever applies)]

Immunoinformatics analysis: IMGT/V-QUEST [or any specific software for NGS-based IG gene analysis (whichever applies)]

**Result:** A productive IGHV3-49*01/IGHD3-9*01/IGHJ4*02 gene was detected. The rearranged IGHV gene showed 100% nucleotide identity with the germline sequence of the IGHV3-49*01 gene.

**Interpretation:** Following the 98% germline identity cut-off which is used for discriminating CLL cases into the IGHV-mutated or IGHV-unmutated category, this case belongs to the IGHV-unmutated category which is generally associated with adverse prognosis. IGHV-unmutated cases exhibit a poor response to chemoimmunotherapy. Moreover, they also experience shorter progression-free survival when treated with fixed-duration treatment that includes venetoclax plus an anti-CD20 antibody compared to IGHV-mutated cases (Al-Sawaf et al. Nature Communications 2023).

Signatures

##### *3. Example of the IG report, IGHV-borderline mutated*

##### Name of the Hospital/Lab

**Determination of IGHV gene SHM status**

Date of result: 22/01/2022

Date of sample collection: 09/01/2022

**Patient name:** ***

Diagnosis: CLL

Tissue type: blood

Molecule type: genomic DNA

**Utilized methodology**

PCR amplification of IGHV-IGHD-IGHJ gene rearrangements with leader primers

Genescan analysis

Bidirectional Sanger sequencing [or next generation sequencing (whichever applies)]

Immunoinformatics analysis: IMGT/V-QUEST [or any specific software for NGS-based IG gene analysis (whichever applies)]

**Result:** A productive IGHV3-49*01/IGHD3-9*01/IGHJ4*02 gene was detected. The rearranged IGHV gene showed 97.3% nucleotide identity with the germline sequence of the IGHV3-49*01 gene.

**Interpretation:** Following the 98% germline identity cut-off value which is used for discriminating CLL cases into the IGHV-mutated or IGHV-unmutated category, this case belongs to the IGHV-mutated category. However, the identity percentage is close to the 98% cut-off and, thus, the case can be considered as borderline-mutated. In such cases, caution is warranted regarding the precise prognostic implications.

Signatures

##### *4. Example of the IG report, Subset #2*

##### Name of the Hospital/Lab

**Determination of IGHV gene SHM status**

Date of result: 22/01/2022

Date of sample collection: 09/01/2022

**Patient name:** ***

Diagnosis: CLL

Tissue type: blood

Molecule type: genomic DNA

**Utilized methodology**

PCR amplification of IGHV-IGHD-IGHJ gene rearrangements with leader primers

Genescan analysis

Bidirectional Sanger sequencing [or next-generation sequencing (whichever applies)]

Immunoinformatics analysis: IMGT/V-QUEST [or any specific software for NGS-based IG gene analysis (whichever applies)]

**Result:** A productive IGHV3-21*01/IGHD: not determined/IGHJ6*02 gene was detected. The rearranged IGHV gene showed 96.8% nucleotide identity with the germline sequence of the IGHV3-21*01 gene.

**Interpretation:** Following the 98% germline identity cut-off which is used for discriminating CLL cases into the IGHV-mutated or IGHV-unmutated category, this case belongs to the IGHV-mutated category. However, this particular rearrangement belongs to stereotyped subset #2 which is associated with adverse prognosis and poor response to chemoimmunotherapy regardless of the somatic hypermutation status (Baliakas et al. Blood 2015; Jaramillo et al. Haematologica 2020). The predictive value of subset #2 for patients treated with targeted agents is currently unknown.

Signatures

##### *5. Example of the IG report, Subset #8*

##### Name of the Hospital/Lab

**Determination of IGHV gene SHM status**

Date of result: 22/01/2022

Date of sample collection: 09/01/2022

**Patient name:** ***

Diagnosis: CLL

Tissue type: blood

Molecule type: genomic DNA

**Utilized methodology**

PCR amplification of IGHV-IGHD-IGHJ gene rearrangements with leader primers

Genescan analysis

Bidirectional Sanger sequencing [or next-generation sequencing (whichever applies)]

Immunoinformatics analysis: IMGT/V-QUEST [or any specific software for NGS-based IG gene analysis (whichever applies)]

**Result:** A productive IGHV4-39*01/IGHD6-13*01/IGHJ5*02 gene was detected. The rearranged IGHV gene showed 100% nucleotide identity with the germline sequence of the IGHV4-39*01 gene.

**Interpretation:** Following the 98% germline identity cut-off value which is used for discriminating CLL cases into the IGHV-mutated or IGHV-unmutated category, this case belongs to the IGHV-unmutated category. Furthermore, this particular rearrangement belongs to stereotyped subset #8 which is associated with the highest risk for Richter’s transformation among all CLL (Rossi et al. Clinical Cancer Research 2009).

Signatures
